# Potential of rice straw biochar, sulfur and ryegrass (*Lolium perenne* L.) in remediating soil contaminated with nickel through irrigation with untreated wastewater

**DOI:** 10.7717/peerj.9267

**Published:** 2020-06-12

**Authors:** Inas A. Hashem, Aonalah Y. Abbas, Abo El-Nasr H. Abd El-Hamed, Haythum M.S. Salem, Omr E.M. El-hosseiny, Mohamed A. Abdel-Salam, Muhammad Hamzah Saleem, Wenbing Zhou, Ronggui Hu

**Affiliations:** 1 Lab of Agricultural Wastes Resource Utilization, College of Resources and Environment, Huazhong Agricultural University, Wuhan, Hubei, People’s Republic of China; 2 Department of Soils and Water Science, Faculty of Agriculture, Benha University, Benha, Qalyubia, Arab Republic of Egypt; 3 MOA Key Laboratory of Crop Ecophysiology and Farming System Core in the Middle Reaches of the Yangtze River, College of Plant Science and Technology, Huazhong Agricultural University, Wuhan, China

**Keywords:** Rice straw biochar, Ryegrass, Nickel, Soil remediation, Sulfur, Untreated wastewater

## Abstract

**Background:**

Untreated wastewater carries substantial amount of heavy metals and causes potential ecological risks to the environment, food quality, soil health and sustainable agriculture.

**Methodology:**

In order to reduce the incidence of nickel (Ni^2+^) contamination in soils, two separate experiments (incubation and greenhouse) were conducted to investigate the potentials of rice straw biochar and elemental sulfur in remediating Ni^2+^ polluted soil due to the irrigation with wastewater. Five incubation periods (1, 7, 14, 28 and 56 days), three biochar doses (0, 10 and 20 g kg^−1^ of soil) and two doses of sulfur (0 and 5 g kg^−1^ of soil) were used in the incubation experiment then the Ni^2+^ was extracted from the soil and analyzed, while ryegrass seeds *Lolium perenne* L. (Poales: Poaceae) and the same doses of biochar and sulfur were used in the greenhouse experiment then the plants Ni^2+^-uptake was determined.

**Results:**

The results of the incubation experiment revealed a dose-dependent reduction of DTPA-extractable Ni^2+^ in soils treated with biochar. Increasing the biochar dose from 0 g kg^−1^ (control) to 10 or 20 g kg^−1^ (treatments) decreased the DTPA-extractable Ni^2+^ from the soil by 24.6% and 39.4%, respectively. The application of sulfur increased the Ni^2+^-uptake by ryegrass plant which was used as hyper-accumulator of heavy metals in the green house experiment. However, the biochar decreased the Ni^2+^-uptake by the plant therefore it can be used as animal feed.

**Conclusions:**

These results indicate that the biochar and sulfur could be applied separately to remediate the Ni^2+^-contaminated soils either through adsorbing the Ni^2+^ by biochar or increasing the Ni^2+^ availability by sulfur to be easily uptaken by the hyper-accumulator plant, and hence promote a sustainable agriculture.

## Introduction

Due to the rapid increase in industrial and urban areas, environmental pollution is increasing worldwide, which is causing unwanted changes in air, water, and soil at biological, physical, as well as chemical levels that ultimately causing negative effects in living things (Nagajyoti, Lee & Sreekanth, 2010; Rehman et al., 2019; Saleem et al., 2020b). In order to feed a growing world population, implemented practices that prevent further contamination and remediate contaminated soils are needed. The agricultural practices in developing countries (arid and semi-arid zones) rely on the irrigation system that complements the scarcity of water and sustain the food production year round (McCartney et al., 2009). At least 20 million hectares of soils in Africa, South America, Middle East, East Asia and Southern Europe are irrigated with untreated sewage water (Bigdeli & Seilsepour, 2008; Qadir et al., 2010).

The discharge of wastewater from industrial and commercial effluents for irrigation purposes led to the accumulation of heavy metals (HM) in the soil and represents a threat for agriculture and food security (Muchuweti et al., 2006; Pedrero et al., 2010; Rageh, 2014). While it is shown that wastewater improves the soil physical properties and increases the soil organic matter content and essential nutrients (Kharche, Desai & Pharande, 2011), it also increases the risk of soil contamination with heavy metals such as lead (Pb), nickel (Ni), cobalt (Co), cadmium (Cd), arsenic (As), mercury (Hg), chromium (Cr) and selenium (Se) (Balkhair & Ashraf, 2016; Mapanda et al., 2005; Rattan et al., 2005; Ullah, Khan & Ullah, 2012), microbes and pathogens (Amoah et al., 2011).

Nickel (Ni^2+^) is one of the common heavy metals used on a large scale in producing metal alloys, stainless steel, ceramic, glass, electronic products and batteries (Rathor, Chopra & Adhikari, 2014). This heavy metal is released in the environment through mining, vehicles exhausts, industrial wastes and applications of fertilizers (Hashim et al., 2017; Kabata-Pendias & Mukherjee, 2007). In order to decontaminate the polluted soils by this heavy metal, chemicals such as acids, chelators, and immobilizers are used which are called in situ Chemo-remediation agents (Abdel-Salam et al., 2015). Many agricultural lands and aquatic ecosystem in Egypt are destroyed or unusable due to the contamination with heavy metals (Al Naggar, Khalil & Ghorab, 2018; Issa, Yasin & Loutfy, 2018). In some areas, crop and fish productions have been reduced by the contaminants.

Soil is the main support of agriculture and plays crucial roles in food safety and security (Tóth et al., 2016). Heavy metal accumulation in soils is of concern in agricultural production due to its adverse effects on food safety and marketability, crop growth, and environmental health of soil organisms (Saleem et al., 2020a). Heavy metals accumulate in soil and crops, and when consumed expose consumers and animals to health hazard (Hashmi et al., 2013). For example, about 20% of the agricultural lands in China are subjected to contamination particularly with heavy metals (Xi et al., 2011), of which Ni^2+^, Cd^2+^ and As^3+^ represent one-fifth of the soil pollution (Wan, Yang & Song, 2018). According to the Chinese Soil Environmental Quality Standards (CSEQS), the level of Ni^2+^ exceeds the tolerance threshold by 4.8% (accentuated by the irrigation with waste water), making it (Ni^2+^) a significant threat to agriculture and land use (Chen et al., 1999; Wuana & Okieimen, 2011). An estimated 6.24% or 137,000 km^2^ of European agricultural lands are destroyed by heavy metals and need urgent remediation (Tóth et al., 2016). A number of previous studies have shown that the Ni ^2+^ contamination of Egyptian soils due to anthropogenic and natural sources endangers the agroecosystem (El-Gammal, Ali & Samra, 2014; Hashim et al., 2017; Nour et al., 2019).

The alleviation of organic and inorganic pollutants level from the soils is a major concern toward protecting the environment and ensuring a sustainable agriculture. Over the past decades, biochar has been developed and promoted as a potential mean to reduce the incidence of manmade pollutants discharged in the environment (Mahdi, El Hanandeh & Yu, 2017b).

Biochar is a complex carbonaceous material produced from pyrolysis of waste biomass and agricultural residues, widely used in water treatment and soil remediation (Liu et al., 2011; Wang et al., 2010; Xu et al., 2013). It is used in agriculture to improve soils fertility, enhance crops yield, and ensure environmental decontamination by sequestrating carbons in the soil for approximately 100–1,000 years (Abiven et al., 2015). Biochar represents a promising choice in chemo-remediation of polluted soil with heavy metals (Lahori et al., 2017). It has unique properties to mitigate contaminants bioavailability due to its tendency to adsorb, immobilize and stabilize heavy metals (Ippolito, Laird & Busscher, 2012; O’Connor et al., 2018).

Rice straw is among the highly available and accessible biomass produced in Egypt. Unfortunately rice straw is burnt after harvesting, thereby causing environmental issues such as air pollution and ecological disturbance (Bishay, 2010). Its availability and accessibility coupled to the necessity of reducing the environmental impacts of its poor management prompted us to use it for producing the studied biochar.

Phytoextraction is a promising, safe and cheap technique for the decontamination of soils polluted by heavy metals which depends upon using plants (hyperaccumulators) to uptake the pollutants from the soil (Ghori et al., 2016). Heavy metals availability in the soil is the main factor which controls the using of this technique successfully. Lowering the soil pH by natural elements such as sulfur (S) is one of the effective ways to increase the availability of heavy metals in soils (Dede & Ozdemir, 2016). Some studies showed that the sulfur addition to mercury-polluted soil reduced the mercury (Hg) uptaken by the plants however, some other studies reported an increasing in the heavy metals solubility due to lowering the pH, therefore the sulfur effect on heavy metals availability in the soil is fuzzy and needs additional studies (Li et al., 2019).

Many recent studies reported that the biochar is effective at reducing heavy metals uptake by plants (Chen et al., 2018; Jatav et al., 2016; Tian, Liu & Xiang, 2017); however, the effect of biochar to remediate Ni^2+^-contaminated soils is fragmentary. Therefore, the main objectives of the present study were: (a) to determine the capacity of the rice straw-biochar in reducing the available Ni^2+^ content in the soil; (b) to illustrate the effect of sulfur in enhancing the Ni-uptake by the hyperaccumulator plant, and finally; (c) to measure the phytoextraction potential of ryegrass—an herbaceous species commonly used as feed for animals and hyperaccumulator of heavy metals—in Ni^2+^-contaminated soils.

## Material and Methods

The study was conducted on nickel-contaminated soil collected from El-Gabal El-Asfar, Qalyubia Governorate, Egypt (Latitude 30°11′38.22″N and Longitude 31°21′56.556″E). The soil at this site is contaminated by Ni^2+^ due to irrigation with wastewater for 40 years. The Ni^2+^-content of the harvested plants from this site contained about 20 folds higher than the maximum critical value of the food safety standard (Gad & Zaghloul, 2007). Two experimental settings were carried out: an incubation experiment and a greenhouse experiment.

### Preparation of experimental materials

#### Soil sampling and analysis

Five hundred kilograms of contaminated soil was surface-sampled at 0–30 cm depth, air dried, crushed and sieved through a two mm sieve. The soil samples were collected from an open field (with no fence) after taking a verbally permission from Diaa-Eldin Elziaty (the owner of the field). The collected samples were mixed for determining its physical and chemical properties before trials using the methods previously reported (Gupta, 2000).

Particle size distribution and calcium carbonate (CaCO_3_) were determined following (Piper, 1950); Electrical conductivity (EC) in saturated soil paste extract was determined following Jackson, Miller & Forkiln (1973) and soil pH was determined in 1:2.5 soil: water suspension (ratio) by using an electronic pH meter (Beckman 350 pH meter, Model N/A, USA) (Jackson, Miller & Forkiln, 1973). Organic matter (OM) content was determined using the Walkley and Black method as described by Jackson, Miller & Forkiln (1973). To determine the total Ni^2+^, the soil samples were digested using tri mixture of perchloric (HClO_4_), nitric (HNO_3_) and sulfuric (H_2_SO_4_) acids (Hseu et al., 2002). The available Ni^2+^ (DTPA-extractable Ni^+2^) was extracted using diethylene triamine pentaacidic acid (DTPA) method (Norvell, 1984). The atomic absorption spectrophotometer 210VGP was used to determine nickel from each treatment. The chemical and physical properties of the studied soil are showed in Table 1. After the physical and chemical analyses, 4 kg of the analyzed soil were introduced in each experimental plastic pot (22.5-cm diameter top, 16.5-cm diameter base and 18-cm depth); these pots were used in the incubation and greenhouse experiments. The pots were padded with plastic bags to prevent water flow out of the pots. Both of the experiments started on October 15th 2018, the incubation experiment lasted for 56 days and ended on December 10th 2018, while the greenhouse experiment lasted for 90 days and ended on January 13th 2019.

**Table 1:** Chemical and physical properties of the studied soil. Each value bar represents means ± standard errors of three replicates.

**Soil property**	**Value**
Particle size distribution^1^
% Sand	82.23 ± 0.04
% Silt	9.58 ± 0.04
% Clay	8.19 ± 0.05
Texture class^2^	Sand
CEC (cmol_c_ kg^−1^)	10.42 ± 0.04
EC (dS m^−1^)	4.1 ± 0.0
pH^3^	6.85 ± 0.02
OM (g kg^−1^)	11.7 ± 0.04
CaCO_3_(g kg^−1^)	15.22 ± 0.03
Total content of Ni (mg kg^−1^)	57.4 ± 0.02
DTPA-extractable Ni (mg kg^−1^)	8.67 ± 0.03

**Notes.**

1 Using pipette method.

2 Texture class is according to international soil texture triangle.

3 Ratio 1:2.5 (soil/water).

### Production of biochar

The biochar was produced from the pyrolysis of rice straw (obtained from the rice producing farmers) in an electrical muffle furnace (Lenton Furnace, UK) under limited oxygen at pyrolysis temperature of 350 °C for 2 h to get high yield of low-pH biochar (Mahdi, El Hanandeh & Yu, 2017b). Then, the resulting product was crushed and milled through 0.25 mm sieve (Khan, Salma & Hossain, 2018) before being applied to the soil. All experiments were carried out in triplicates. The chemical and physical properties of the biochar are presented in Table 2.

**Table 2:** Properties of the rice straw biochar. Each value bar represents means ± standard errors of three replicates.

**Property**	**Value**
pH^a^	7.08 ± 0.02
EC (dS m^−1^)	1.28 ± 0.02
CEC (cmol_c_ kg^−1^)^b^	64.2 ± 0.03
Available Ni mg kg^−1^^c^	nd

**Notes.**

a Determined in 1:2 (w/v) suspension.

b Sumner & Miller (1996).

c Measured in the ash.

nd not detected

### Experimental designs

#### Incubation experiment

Factorial randomized complete block design (RCBD) was used for this experiment. Five incubation periods (time elapsed between the adding of biochar to soils and the analysis of nickel content) (1, 7, 14, 28 and 56 days) were considered and three biochar doses (amounts applied) (0, 10 and 20 g kg^−1^ of soil) were used under fluctuating greenhouse conditions (temperature of 15 ± 5 °C, and relative humidity of 50 ± 8%). The untreated groups (0 g kg^−1^) represent the control. In order to increase the availability of Ni^2+^ in the soil, we used two doses of elemental sulfur (amounts applied) (0 and 5 g kg^−1^ of soil), which was commonly used in lowering the pH of the soil and increasing heavy metal availability (Komkiene & Baltrenaite, 2016). Overall, the experiment involved 30 treatments, each consisting of three replicates. A total of 90 pots were used, each containing 4 kg of the soil treated with different doses of biochar (0, 10 and 20 g kg^−1^) and sulfur (0 and 5 g kg^−1^). Tap water (0 Ni^2+^ mg L^−1^) was supplied continually to keep the moisture content of the soil at the water holding capacity by weight of each pot. At the end of each incubation period, the Ni^2+^ was extracted and analyzed using Diethylene Triamine Pentaacetic Acid (DTPA) method as previously described (Lindsay & Norvell, 1978).

#### Greenhouse experiment

A factorial randomized complete block design (RCBD) was used. Similar to the incubation experiment, three doses of the biochar and two doses of the sulfur were used. The pots were uniformly packed with 4 kg of the soil, treated with different doses of biochar and sulfur (see incubation experimental procedures above). Thirty seeds of ryegrass were sown in each pot and were allowed to germinate and grow under the greenhouse conditions (15 ± 5 °C, 50 ± 8% relative humidity). Pots were watered using tap water (0 Ni^2+^ mg L^−1^) as required to keep the moisture content at water holding capacity. After germination, the emerged seedlings were thinned to 20 plants per pot based on their size, shape and color of the leaves. The grown plants were supplied with the essential nutrients Nitrogen, Phosphorus, Potassium (N-P-K) 300: 100: 200 (mgL^−1^) respectively, through foliar application once per week. The grass was harvested twice; the first mow was on the 45th day of the cultivation, while the second one was on the 90th day. The harvested plants were oven-dried at 70 °C for 72 h, then crushed, milled through a 1-mm stainless steel mill and digested according to a previously described method (Grimshaw, 1987). The Ni^2+^ content of the plants was determined using atomic absorption spectrophotometer 210VGP (Buck Scientific, USA).

### Statistical analyses

All data on nickel immobilization and uptake were tested for homogeneity of variances using Levene’s tests. The important factors (treatments, incubation periods and mow time) that influenced the Ni^2+^ adsorption and uptake were evaluated by the regression analysis (SPSS) with treatments, incubation periods and mow time as factors. The one-way analysis of variance (ANOVA) was used to analyze differences in Ni^2+^ adsorption and uptake across treatments and experimental periods using SPSS 20.0 software (Statsoft Inc, Carey, J, USA). Tukey Post-hoc (HSD) test was used for mean separations within and between different treatments and experimental times. Differences between the Ni^2+^ immobilization capacities of the biochar and uptake levels were expressed as the means with standard errors (SE) and were considered significant when the *P* values were less than 0.05 after comparison with Tukey Post-hoc (HSD) test. OriginPro software version 8.5.1 was used to draw figures.

## Results

### Immobilization of nickel by biochar

**Figure 1:**
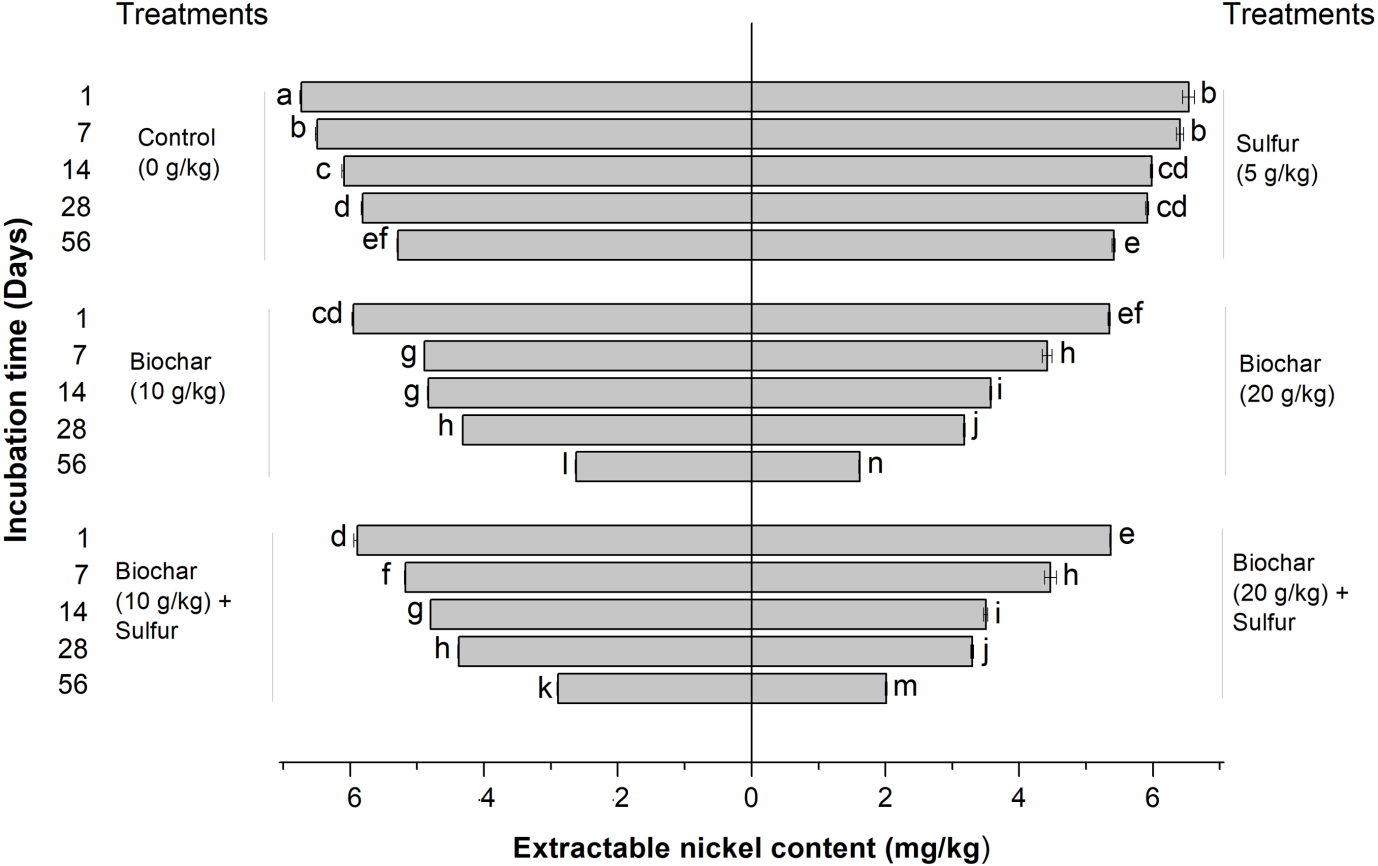
Effect of biochar and sulfur doses on the DTPA-extractable nickel contents of the contaminated soil. Each vertical bar represents means ± standard errors of three replicates, and means followed by lowercase letters within and between treatments are statistically different after Tukey HSD test at *P* = 0.05.

Effect of biochar and sulfur doses on the DTPA-extractable nickel contents of the contaminated soil is shown in Fig. 1. Figure 1 indicates that the biochar treatments (alone and in combination with sulfur) and the experimental period significantly affected the extractable Ni^2+^ content of the soil following a dose-dependent pattern in comparison with the control groups (untreated) (Regression Model, *F* = 8.926; *df* = 5, 12; *R*^2^ = 0.788; *P* = 0.001, and *F* = 143.913; *df* = 6, 8; *R*^2^ = 0.991; *P* < 0.001, respectively, Fig. 1). The extractable Ni^2+^ content of the soil reflected the extent of Ni^2+^ immobilization by biochar. The lower the extractable Ni^2+^ content of the soil, the heavier is the immobilization of Ni^2+^ by biochar. When treated alone, the sulfur did not produce any effect on the Ni^2+^ immobilization compared with the control groups (ANOVA, *F* = 8.926; *df* = 1, 4; *P* = 0.079, Fig. 1). When treated by sulfur combined with the biochar, the extractable Ni^2+^ content was not significantly different from those of single biochar treatments (ANOVA, *F* = 8.926; *df* = 1, 4; *P* = 0.912 and *F* = 8.926; *df* = 1, 4; *P* = 0.999, respectively, Fig. 1). The Ni^2+^ immobilization level increased based on the doses of biochar used. The least content of extractable Ni^2+^ among all the treatments was recorded when the soil was treated with 20 g kg^−1^ of single biochar treatment (ANOVA, *F* = 1612.095; *df* = 1, 4; *P* < 0.001, respectively) (Fig. 1).

### Effects of biochar on nickel uptake by plants

**Figure 2:**
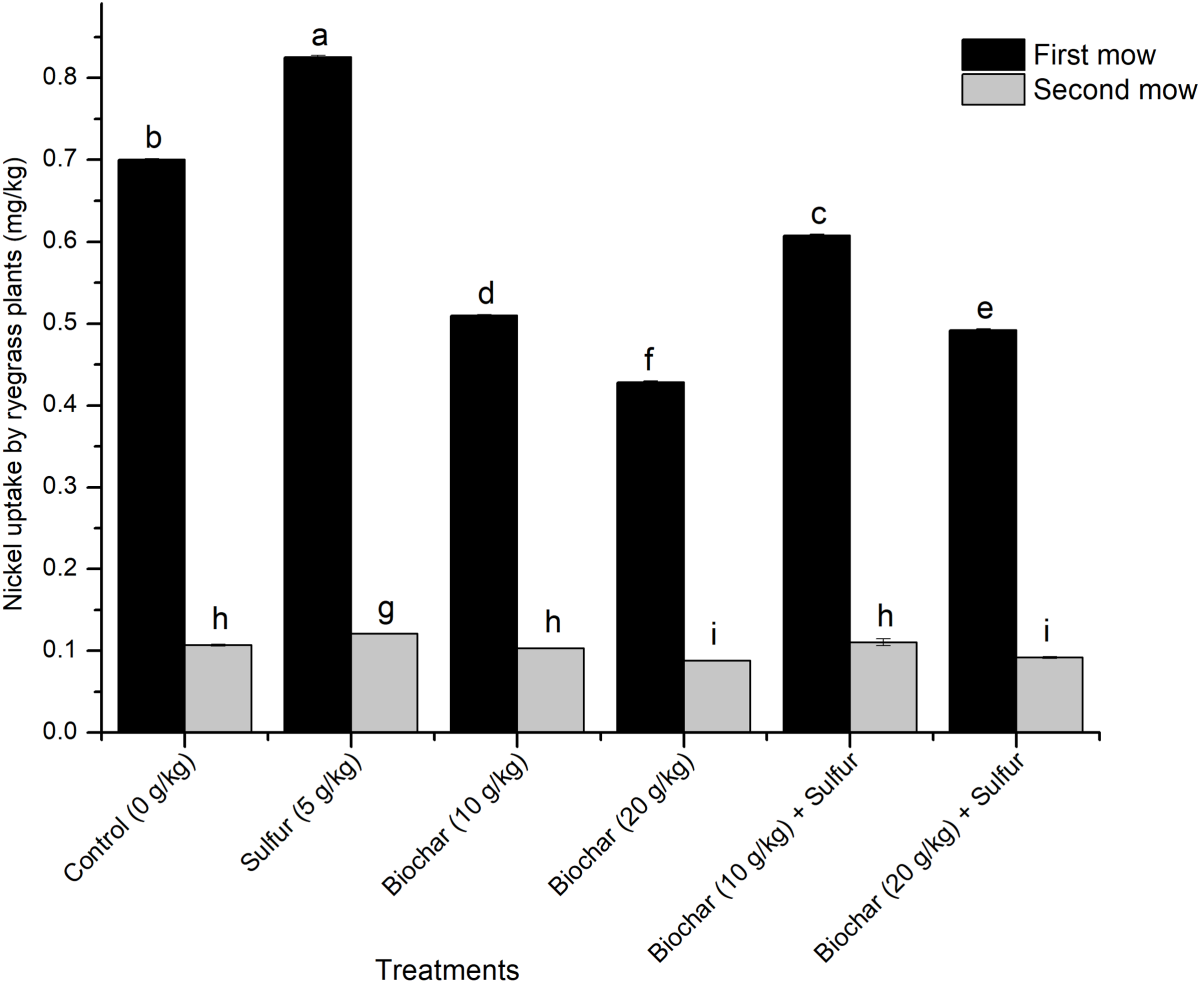
Effect of biochar and sulfur doses on nickel uptake by ryegrass plants. Each vertical bar represents means ± standard errors of three replicates, and means followed by lowercase letters within and between treatments are statistically different after Tukey HSD test at *P* = 0.05.

Effect of biochar and sulfur doses on nickel uptake by ryegrass plants is shown in Fig. 2. Figure 2 shows that the Ni^2+^ uptake by the ryegrass was significantly influenced by the experimental treatments and mow periods (Regression Model, *F* = 11.078; *df* = 2, 15; *R*^2^ = 0.596; *t* = 3.058; *P* = 0.001, and *F* = 11.078; *df* = 2, 15; *R*^2^ = 0.772; *t* = 3.058; *P* = 0.008, respectively). The single application of sulfur enhanced the Ni^2+^ uptake compared to the control (ANOVA, *F* = 9360.151; *df* = 5, 12; *P* < 0.001) (Fig. 2). The Ni^2+^ uptake was significantly higher in the first mow in comparison with the second one (ANOVA, *F* = 9360.151; *df* = 5, 12; *P* < 0.001) which was consistent across the treatments (there were no treatment effects on the Ni^2+^ uptake in the second mow) (ANOVA, *F* = 44.252; *df* = 5, 12; *P* = 0.058) (Fig. 2). Both doses of biochar mitigated the Ni^2+^ uptake by the plant compared with the control (ANOVA, *F* = 9360.151; *df* = 5, 12; *P* < 0.001) (Fig. 2). The blend of biochar with 5 g kg^−1^ of sulfur increased the uptake of Ni^2+^ by the plant (in the first mow), compared to their single applications (ANOVA, *F* = 9360.151; *df* = 5, 12; *P* < 0.001).

## Discussion

In the present study, we used rice straw to produce the biochar and then evaluated its efficiency in the mitigation of Ni^2+^ contamination from Ni^2+^ contaminated soil and how it triggered the Ni^2+^ uptake by ryegrass plant (*Lolium perenne* L.). Overall, the biochar used in our experiment exhibited a good alleviation performance of soil Ni^2+^ contamination and mitigation of Ni^2+^ uptake by the plants.

### Mitigation of Ni^*t*2+^ contamination in the soil by biochar

The results obtained from this experiment show that the DTPA-extractable Ni^2+^ content of the soil decreased with the increase of the biochar doses applied, reveal that increasing the dose of biochar resulted in enhancing the Ni^2+^ adsorption by the biochar (Fig. 1). These results join previous reports (Ali et al., 2020b; Mahdi, Qiming & Hanandeh, 2018; Pedrero et al., 2010; Rageh, 2014) in establishing the capacity of biochar to alleviate the content of heavy metals from contaminated soils. For examples, rice straw biochar has proven its efficiency in alleviating Ni^2+^ toxicity and remediating Ni^2+^-contaminated soils by decreasing the Ni^2+^ mobility and leachability in the soil (Ali et al., 2020a; Ali et al., 2020b), also date seed derived biochar has shown significant capacity to adsorb copper (Cu^2+^) and Ni^2+^ ions from aqueous solution, and the ions removal depended on the pyrolysis temperature and time used in biochar preparation and the biochar dose (Mahdi, El Hanandeh & Yu, 2017a; Mahdi, El Hanandeh & Yu, 2017b), moreover the biochar produced from wood waste revealed significant potential of Cu^2+^ adsorption from the soil as well the Cu^2+^ adsorption quantity increased with the increase of the biochar doses and pH value (Tomczyk, Boguta & Sokołowska, 2019). In other studies, it was previously reported that the adsorption capacity of biochar is related to its pH and cation exchange capacity (CEC) values. The high CEC value, the large surface area and the alkaline pH of biochar could explain its potentials to adsorb and immobilize pollutants from soils (Ali et al., 2020a; Beesley et al., 2011; Jeffery et al., 2011; Kookana, 2010; Yuan & Xu, 2011), owing to the fact that the alkali pH results in the functional groups dissociation of biochar, these functional groups such as phenolic and carboxylic groups produce negative charge thereby easily immobilize the soil cations which have positive charge (Tomczyk, Boguta & Sokołowska, 2019). Additionally, the biochar efficacy in heavy metal adsorption, stabilization and precipitation was attributed to its large surface area and complexation between the functional groups and the metals (Ali et al., 2020a; Lu et al., 2012). Furthermore, it has been suggested that heavy metals such as Ni^2+^ and Cd^2+^ were immobilized by the biochar due to its porous structure and the existence of several functional groups and negative charges on its surface (Ali et al., 2020b; Kamran et al., 2019).

The role of the sulfur is to lower the soil pH with the effect of increasing the availability of Ni^2+^ in soils (Dede & Ozdemir, 2016). This will in turn, enhance the biochar adsorption capacity or the Ni^2+^ uptake by the hyper-accumulator plant. The availability and motions of heavy metals increase in low pH conditions of the soil (Çimrin, Turan & Kapur, 2007; Dede & Ozdemir, 2016) also, the Ni^2+^ content released from river sediments decreases with the increase of the water pH (Zhang et al., 2018). The results of the incubation experiment have shown no statistic difference of Ni^2+^ adsorption between the control (untreated) groups and the sulfur treatment groups under pH (6.85), irrespective of the incubation time. However, the results of the greenhouse experiment revealed that the single application of sulfur significantly increased the Ni^2+^ uptake by ryegrass plant. This finding indicates that the sulfur might not have been able to significantly decrease the soil pH in view of releasing more nickel ions to be adsorbed by the biochar in the incubation experiment (as occurred in the greenhouse experiment). This may be due to differences in the incubation and cultivation conditions in the laboratory and greenhouse, respectively. Moreover, using the elemental sulfur to lower the soil pH is a slow biological process, instead of a fast chemical reaction. This biological process relies on (a) the potential of soil microorganisms such as sulfur-oxidizing bacteria and fungi (which are abundantly available in the rhizosphere area) to oxidize the elemental sulfur (S) to sulfate (SO_4_^2−^), which quickly turns into sulfuric acid (H_2_SO_4_) to reduce the soil pH (Gao & Draper, 2010; Grayston & Germida, 1991); (b) the soil temperature and humidity. The sulfur-oxidizing bacteria need warm and moist soil to be active and play its oxidizing role (Gao & Draper, 2010). In fact, soils have the ability to absorb the heat in sunny days and store the thermal energy due to its large heat storage capacity (Alnefaie & Abu-Hamdeh, 2013; Rempel & Rempel, 2013);, and finally, (c) the roots exudates. It was shown that the roots secretions in the rhizosphere area improve the biological processes and the microbial community (Kuzyakov, Hill & Jones, 2007), thereby enhancing the sulfur oxidation process and lowering the soil pH. The sulfur inability to lower the soil pH in the incubation experiment can also be attributed to the sulfur dose used (5 g kg^−1^), which might not have been enough to lower the soil pH within the incubation time. A higher sulfur dose of 9.6 g kg^−1^ previously applied was reported to be able to decrease the pH of the soil and increase the solubility of heavy metals (Pb^2+^ and Cd^2+^) (Çimrin, Turan & Kapur, 2007). Another possible mechanism linked to the application of sulfur could be the increase of the transpiration rate, which in turn, might have increased Ni^2+^ translocation to shoot through water movement. In our study, we did not measure transpiration rate of ryegrass, but it’s believed that it is affected by the application of sulfur (Habiba et al., 2015; Kanwal et al., 2014; Zaheer et al., 2015). Therefore, the evaluation of the transpiration rate of ryegrass represents a tangible venue of our future research.

Furthermore, the addition of sulfur to the biochar did not significantly affect the content of extractable Ni^2+^ as no significant difference of its content was recorded compared to single application of biochar (Fig. 1). Presumably, the absorption or immobilization efficiency of Ni^2+^ by biochar was significantly triggered, rather than that in the presence of sulfur in the combined treatment.

### Reduction of the Ni^2+^ uptake of ryegrass by biochar

Perennial ryegrass (*Lolium perenne* L.) is an herbaceous plant species commonly used as feed for animals and as hyper-accumulator of heavy metals from soils (Zou, 2015). In the greenhouse experiment, the Ni^2+^ uptake capacity by ryegrass was evaluated under the influence of biochar and sulfur. Overall, the results show that the ryegrass uptake of Ni^2+^ was lower at the first mow in biochar treatments and the uptake capacity was inversely proportional to the doses of biochar used in comparison with the control (Fig. 2). The reduction of the Ni^2+^ uptake by the plant after the first mow suggests that the biochar could have adsorbed most of the available Ni^2+^ ions in the soil thereby reducing its amount to be up-taken by the plant. This result is supported by some previous reports whereby, the addition of biochar to HM-contaminated soils decreased the availability of Ni^2+^, Pb^2+^, Cd^2+^ and Cu^2+^, ensured optimal uptake by maize plant and prevented a potential phyto-toxicity (Alaboudi, Ahmed & Brodie, 2019; Kamran et al., 2019; Rehman et al., 2019; Rehman et al., 2016).

The highest Ni^2+^ uptake was recorded when the soil was treated with sulfur alone (5 g kg^−1^ of soil) with 17.58% of Ni^2+^ up-take increase compared to the control. As explained in the previous section, the decrease of the pH by the application of sulfur resulted in the increase of the availability of Ni^2+^ ions, which thereafter, augmented the Ni^2+^ uptake efficiency of the plant compared to the control (Fig. 2). In previous studies, the application of sulfur significantly increased the removal or uptake of Cu^2+^, Pb^2+^ and Cd^2+^ ions by the plants in consequence of increasing the ions solubility due to the pH reduction (Çimrin, Turan & Kapur, 2007; Dede & Ozdemir, 2016). Therefore, the sulfur can be used in phytoremediation to raise the plant potentials in heavy metals extraction and uptake.

The Ni^2+^ uptake was lower in the second mow compared to the first one and remained consistent across treatments. This observation indicates that the Ni^2+^ uptake process could have been carried out at the early stage of the development of the ryegrass (within 45 days). It seems like as the plant grows older, its Ni^2+^ uptake capacity crashed and remained below the uptake threshold (less than 0.1 g kg^−1^) (Fig. 2), in addition to the reduction of the soil available Ni^2+^ content caused by biochar application. Our finding is consistent with a study on decontamination of Ni^2+^-contaminated soils collected from different locations of China, in which *Alyssum corsicum* and *Alyssum murale* plant species showed a very low Ni^2+^ uptake in Yuanjiang soil (Qiu, Liu & Wan, 2008). The fact that the Ni^2+^ ions were significantly up-taken during the first mow (on the 45th day of the cultivation) and dropped consistently in the second mow (on the 90th day of the cultivation) suggests that the biochar could reduce the level of available Ni^2+^ in the soil and prevent its uptake by the plant; and the ryegrass could be used as a hyper-accumulator of heavy metals to clean the soil (preferentially at the early stage of its development) and as animal feed (at the late stage of its development). This dual benefit could help the farmers to increase their crops (by decontaminating the soil from pollutants) and to empower the livestock industries by availing safe feed with very low contamination rate.

## Conclusions

Biochar is considered as a promising adsorbent in chemo-remediation, it’s cost-effective and eco-friendly. In this study, we performed two experimental settings (incubation and greenhouse experiments) to investigate the mitigation effect of Ni^2+^ contaminated soil by application of rice straw biochar and how it triggers the Ni^2+^ uptake by ryegrass. The results show that the mitigation effect of Ni^2+^ contamination by biochar is dose-dependent therefore it can be used to reduce the level of Ni^2+^ in the soil and its uptake by the plants. The single application of sulfur increased the Ni^2+^ uptake by ryegrass due to increasing the Ni^2+^ availability by lowering the soil pH, contrary to its combined application with biochar, in which the Ni^2+^ uptake by the plant decreased, therefore, the sulfur can be used in phytoremediation to raise the heavy metals uptake by the plants, and the ryegrass could be used as a hyper-accumulator of heavy metals (at the early stage of its development) and also as animal feed (at the late stage of its development), thereby promoting a sustainable agriculture.

##  Supplemental Information

10.7717/peerj.9267/supp-1 Data S1 Incubation and the greenhouse experiment raw data

## Additional Information and Declarations

### Field Study Permissions

The following information was supplied relating to field study approvals (i.e., approving body and any reference numbers): This research did not involve any animal or human as guinea-pigs. The soil samples were collected from an opened field (with no fence) irrigated by wastewater for 40 years. The owner of the field verbally allowed us to collect soil samples to conduct our research. The permission was verbally given by Diaa-Eldin Elziaty.

### Data Availability

The following information was supplied regarding data availability: The raw measurements are available in the Supplementary Files.
